# Aging Veterans and a Silver Lining of Service

**DOI:** 10.21061/jvs.v9i3.495

**Published:** 2023-12-20

**Authors:** Traben Pleasant, Sarah Ono, Sarah Gothard, Rachel Wall, Lisa Silbert

**Affiliations:** Department of Veterans Affairs, 3710 SW U.S. Veterans Hospital Road, Building 6, Portland, OR., 97239-2999; (503) 220-8262 x57766, US; Department of Veterans Affairs, 3710 SW U.S. Veterans Hospital Road, Building 6, Portland, OR., 97239-2999, US; Oregon Center for Aging & Technology, Oregon Health & Science University, Portland, Oregon, US; Department of Veterans Affairs, 3710 SW U.S. Veterans Hospital Road, Building 6, Portland, OR., 97239-2999, US; Aging and Alzheimer’s Disease Center, Department of Neurology, Oregon Health and Science University, Portland 97201, US

**Keywords:** aging, military-related disabilities, post-9/11, veterans, Vietnam

## Abstract

The objective of this study was to explore aging veteran’s military experiences, including serving in conflicts or wars and their military-related health issues, with a focus on the impacts of their experiences on the aging process. A cohort of 48 Pacific Northwest, primarily rural, Vietnam-era veterans responded to a survey questionnaire emailed in 2021. The main survey question addressed in this article is, “Do you believe that your military experience has made aging more difficult?” Fifty percent of this cohort served in a conflict or war, mostly in Vietnam, and 68% reported having military-related health issues. We used veterans’ survey responses to create this article which is a hybrid narrative—a mix of poetry and prose. Regardless of serving in conflicts or wars and their military-related health issues, most veterans found a silver lining of service that acts as a source of pride and resilience that is beneficial to post-military life as they age.

Compared to traditional academic writing, poetry offers a vulnerable vibrancy as an uncommon and creative form of research dissemination with a unique reach and resonance (Miller, 2018; Rajabali, 2014). The poem below is based on survey data collected and analyzed by our research team regarding the lived experience of aging veterans. Our team, from the Department of Veterans Affairs (VA), Portland, Oregon, and the Oregon Health and Science University (OHSU), was comprised of three anthropologists, four analysts, and an expert in neurology. We sought to address the following research question: “Do you believe that your military experience has made aging more difficult?” Our cohort of Pacific Northwest, primarily rural, aging Vietnam-era veterans were enrolled in the Collaborative Aging Research using Technology Initiative (CART; IRB 4089). CART provides valuable insights into cognitive decline and aging using technology (passive sensors) while participants age in place at home (Beattie et al., 2020). In 2021, our survey was emailed to 120 CART participants. Our final sample comprised of 48 Vietnam-era and a few post-9/11 veterans. Of the final 48 participants, 45 identified as male and 3 identified as female, with an average age of 73.8. Also, from this sample, 50% served in a conflict or war, with 68% reporting military-related health issues. We found that 71% of the participants said no, that their military experience did not make aging more difficult, while 23% said yes, it did make aging more difficult, and 6% did not respond. Veterans’ responses were used to create our semi-rhythmic narrative using poetry and prose.

Regardless of conflicts, war, and health issues, most veterans find a silver lining of service, like a “glistering guardian, if need were to keep [veteran] life and honour unassailed” (Milton, 1634/1799, **p. 22**). This silver lining of service that remains can be beneficial throughout a veteran’s life as a kind of lasting honor and resilience gained from one’s military service (Lee et al., 2017; Spiro III et al., 2016). According to most veterans in our study, military experiences, including mental and physical trauma, have not made aging more difficult. Their insights help us to better understand rural and aging veteran perspectives and the implications for younger yet aging post-9/11 veterans with similar experiences and perhaps with similar futures.

If the military is connected to a veteran’s trauma or pain,

does a silver lining of service extinguish any military disdain?

As an aging post-9/11 veteran of the Iraq War,

every year, I feel each bump, blast, boom, and beer more and more.

Proud of my service, critical of war, I wonder, “What does it all mean?”

I think, “Woah the war was real?” holding my daughter’s hand while in line for an ice cream.

Our research team wondered what older veterans perceived as typical mental and physical pain.

So we sent a survey asking, “Do you believe that your military experience has made aging more difficult?” Please explain.

Our participants were enrolled in a multisite, nationwide study we call CART.

Using passive sensors they try to detect cognitive decline, monitoring gait, sleep, driving, and probably art (Beattie et al., 2020).

Veterans were vetted to see who could be included.

If you weren’t at least 62, living independently or with a partner, or couldn’t chat via email you were excluded.

Also excluded were those with dementia or illness that would prevent the study from going on.

To examine gait changes, excluded too were veterans who were not able to walk, in their homes.

We had 48 participants, 45 males, 3 females, on average high school educated and age 74.

Mostly White and married, but a good chunk of veterans had also been divorced.

No Space Force service seen,

these veterans represented the Army, Navy, Coast Guard, Air Force and Marines.

Over half of our plucky participants served in a conflict or a war, or two,

mostly Vietnam, but the Persian Gulf, Afghanistan, and Iraq Wars were experienced by a few.

We asked about their mental and physical trauma experienced during their service.

Raucous environments, training accidents, and the Vietnam War should make you nervous.

Seventy percent reported a military-related disability,

often referred to herein as an MRD.

MRDs were reported by mostly those who had served in a conflict or war,

but nearly a third of those who never saw a war, MRDs their bodies still bore.

Was it due to an accident, a blast, or crash we asked without much zig or zag.

“Yes,” a third said, so we all should complain less when the French Fries aren’t in the bag.

WAR no, PEACE yes, unless

WAR means All Day Music or Slippin’ Into Darkness

Cut on The End by the Doors.

This is what you signed up for.

This is the end,

my only friend, the end.

Artillery booms, mortar blasts, and grenades exploding nearby… Bang!!!

Shooting and getting shot at under flaming helicopters and soon-to-be downed planes.

Veterans suffer from lost friends; it’s “A Love Supreme” for those forever deployed.

They also suffer from Agent Orange, back and knee injuries, and hearing loss due to noise.

Veterans reported Traumatic Brain Injuries, with some living undiagnosed to boot.

And a third of this cohort reported suffering from mental health-related issues too.

Most had PTSD and some felt they were living undiagnosed as well.

Oh, what heaven it would be to do it all again, oh to go through hell.

My great-uncle Murphy Pleasant Jr. died in Vietnam at 20 years, 11 months, 22 days young.

Please leave a flower for him at the Vietnam Veteran Memorial Wall, Panel W35, Line 21 (The Virtual Wall, 2019).

Forty percent of our veterans have sleep issues, which can be quite scary when out of control (Hughes et al., 2018; Ryden et al., 2019). Insomnia was reported to be directly related to the military service of half of these tired souls.

But how do you know what or who to blame as military pain unfolds?

Was it the blasts, the blood, the bugs, the back-breaking labor, or are you just getting old?

Our central question for these veterans regarding their perspectives on aging normal versus atypical was, “Do you believe that your military experience has made aging more difficult?”

Aging more difficult you say? Twenty-three percent of veterans said *YES* that it did in fact.

Most of them had physical or mental MRDs, some served in Vietnam and a couple in Iraq.

A veteran’s life can be tough, and some may still feel sad or mad or mad at being sad.

The military plus aging can mean difficulty for them, and here are a few reasons they had:

Years on the ocean, and climbing and bracing for impacts constantly.

Knee pain and increasingly decreasing mobility.

Exposure to Agent Orange and the impact of living in a war zone.

An acute awareness of life’s fragility, feeling old, anxious, alone.

Memory problems and difficulty multitasking.

A poor sense of direction and hearing loss from explosions, huh…what were you asking?

One veteran said that it was a hard question to answer, which is fair,

because aging, he said, inherently required more medical care.

Do you believe that your military experience has made aging more difficult? Seventy-one percent said *NO*, it hadn’t.

Nearly half of this group saw Vietnam, and many suffered from military-related damage.

In light of trauma, their results are interesting and should give you a clue.

Aging veteran perspectives are very dynamic, and they speak to non-veterans too (Richard-Eaglin et al., 2020).

Many of these old veterans felt they were doing just fine aging.

Some reported no issues at all, but careful, veterans can be stoic, tough, and avoid complaining.

In their youth, these vets were top-notch service members in peak mental and physical fitness.

Then they experienced dire combat and the loss of life in contexts I hope you never witness.

Here are some of the silver linings embedded in their survey stories.

Veterans grew stronger through hard times similar to *Apocalypse Now, Restrepo*, and *Glory*.

A humorous and philosophical response received makes me want to explore aging more.

A veteran summarized that they couldn’t say if the military had made aging more difficult, for they had not been that age before.

We must look forward as veterans and our general society grows older,

to post-9/11 veterans and the national and global aging booms healthcare systems will shoulder.

Post-9/11 veterans are part of the most female-populated, racially diverse US military ever.

They have higher rates of MRDs than veterans of previous military eras (US Census, 2020).

Healthcare should be imaginative, clever, efficient, and careful to prep for aging blooms.

So, by an increased reliance on healthcare services, our systems are not consumed (Ogura & Jakovlijevic, 2018).

Crunchy-knee older veterans use VA healthcare more than the young (US Census, 2020).

Nothing hurt back then in our youth, still drunk from the night before while on morning runs.

But what if you come of age in the military, in a conflict or war, what persists?

Based on this study and others, a silver lining of service remains for most who enlist.

However, this mostly Pacific Northwest, White-male Veteran cohort has limits you see.

Their experiences may not generalize to veterans from different regions, classes, and ethnicities.

Still, 18 years old or 80, a pawn or a player,

our collective identity as US military veterans is the bond that we share.

A complex type of honor, courage, and commitment that does not die with age.

The military seems to build a lasting resiliency, so against the dying of the light we rage.

As we age and reflect, glistening thin and bright around clouds of any military service disdain, for most veterans, a silver lining of service still remains.
